# Examining Implicit Attitudes towards Exercisers with a Physical Disability

**DOI:** 10.1155/2013/621596

**Published:** 2013-04-04

**Authors:** Cassandra D. Dionne, Heather L. Gainforth, Deborah A. O'Malley, Amy E. Latimer-Cheung

**Affiliations:** School of Kinesiology & Health Studies, Queen's University, 28 Division Street, Kingston, ON, Canada K7L 3N6

## Abstract

*Background.* Using measures of explicit attitudes, physical activity status has been established as a factor that reduces the stigma able-bodied people hold towards people with physical disabilities. This phenomenon is called the exerciser stereotype. However, whether the exerciser stereotype exists when using measures of implicit attitudes remains unknown. *Objective.* The aims of this study were to evaluate the prevalence of negative implicit attitudes towards people with physical disabilities and determine whether implicit attitudes towards people with physical disabilities were influenced by the exerciser stereotype. *Methods.* One hundred able-bodied participants (82 females, 18 males) completed two implicit association tests (IATs): the Disability-Attitudes IAT and the Disability-Activity IAT. The Disability-Attitudes IAT measured implicit attitudes towards people who were not disabled relative to disabled; the Disability-Activity IAT measured attitudes towards people with a physical disability who were active relative to inactive. *Results.* Results revealed that 83.8% of participants had negative implicit attitudes towards people with a disability. Participants held more positive attitudes towards active versus inactive people with a physical disability. *Conclusions.* The study findings indicate that the exerciser stereotype exists implicitly and may undermine negative attitudes towards people with physical disabilities.

## 1. Introduction

Stigma has been defined as an adverse reaction to the perception of a negatively evaluated difference [1]. From this definition, stigma can be understood as a broad term that incorporates three elements: problems of knowledge (ignorance), problems of attitudes (prejudice), and problems of behaviour (discrimination) [2]. Stigma accompanies a variety of health conditions, including individuals with physical disability [3]. Unfortunately, the stigma people with physical disabilities experience can have negative effects including psychological stress, depression, fear, participation restrictions, and increased risk of disability and advanced disease [3]. It is important to better understand stigma to reduce the occurrence of these negative effects. To understand this stigma, however, it is necessary to understand the attitudes that able-bodied people hold towards people with a physical disability. Measuring these attitudes is an important first step in understanding the stigma that people with a physical disability experience. 

### 1.1. The Dual Processing Theory: Implicit and Explicit Attitudes

Investigating stigma can prove to be difficult as able-bodied people are aware that they should have egalitarian attitudes and behaviours towards persons with physical disabilities [4]. Furthermore, studies that have been conducted on stigma and attitudes have found that people often behave in ways that appear to be inconsistent with their feelings in the presence of those who are stigmatized [5]. Looking at physical disabilities from this perspective means that a person may hold negative attitudes towards people with a physical disability, but their actual behaviour may reflect sympathy and kindness [5]. A good example of this inconsistency was demonstrated in a study by Kleck [6] who found that, when asked to teach origami (paper folding) to a person in a wheelchair, able-bodied people indicated that their impressions of the individuals with a physical disability were very positive. However, the nonverbal behaviour of those teachings indicated anxiety and avoidance, such as sitting farther away from the individual [6, 7]. Therefore, there was a discrepancy between the teachers' more controlled, verbal communication and their less controlled, non-verbal communication [5]. 

The discrepancy between more controlled verbal communication and less controlled, non-verbal communication reflects the idea that people may hold negative attitudes towards a person with a disability, but appear to hold positive attitudes in their presence. This discrepancy can occur because people process information on both an explicit and implicit levels [8]. 

Explicit attitudes are conscious, controlled, and reflective, whereas implicit attitudes are defined as attitudes that exist without any conscious awareness of the respondent [9]. These two types of attitudes can be well understood by looking at dual-processing theories, which have been developed by social psychologists in order to understand phenomena such as prejudice and stigma [5]. 

Although there are a variety of different dual-processing theories proposed [5, 10–13], at the core of all theories is the explanation that there are two psychological methods by which people process information: associative processing and rule-based processing [5]. Associative processing is how implicit attitudes are formed. Attitude formation occurs through automatic affective reactions (i.e., immediate emotional reactions [5]). According to Fazio and Olson [14], merely being exposed to a stigmatized person immediately brings to mind negative evaluations. The perceiver need not intend for these reactions to occur and need not invest any conscious effort to produce them; they are automatic. In contrast, rule-based processing is how explicit attitudes are formed. This type of processing involves conscious, deliberative, and thoughtful reactions [5]. Consequently, explicit attitudes may be subject to social desirability effects, which is the tendency to respond in a manner thought to be favourably viewed by others [9]. As a result, explicit attitudes may not always reflect a person's true feelings. 

Based on how explicit attitudes are formed, it is not surprising that explicit attitudes are a good predictor of behaviour that is intentional and under conscious control, such as friendliness. Implicit attitudes, on the other hand, have been found to better predict actual discrimination behaviour because these attitudes are not susceptible to social desirability [9]. Researchers studying stigma towards people with physical disabilities such as an SCI should consider the limitation of social desirability with explicit attitudes and ensure that they are measuring actual stigma and not simply what people believe are socially acceptable attitudes. Therefore, the present study measured implicit attitudes in order to avoid social desirability effects, which can occur when measuring explicit attitudes. 

### 1.2. Measuring Implicit Attitudes

One of the most popular current methods of measuring implicit attitudes is the Implicit Association Test (IAT) [4, 15]. The IAT measures implicit attitudes by enabling the researcher to assess how quickly a person can classify words or pictures into target categories [4]. Because the IAT utilizes speed of response, it is a measure of implicit attitudes, not explicit attitudes which may be subject to social desirability effects. Assessing categorization speed is a quantitative way of measuring differences in the association between target concepts and evaluative attribute categories (usually good and bad). It is possible to measure the differences of these associations based on the principle that it should be easier to respond to concepts that are congruent to your way of thinking than to concepts that are incongruent [16]. This method thereby measures the underlying automatic evaluation of positively or negatively associated words or symbols [15]. 

There are two variations of the IAT. The first is the original, which was created by Greenwald et al. [15] and uses a computer to measure participants' response latency when presented with target stimuli and valence words. The second is a paper-pencil version, which, instead of response latency, measures the number of items in each block that has been correctly categorized in 20 seconds [4, 17]. The paper-based method has been found to be a useful measure of individual and group differences in implicit attitudes [4, 17–20] and has the added benefit that it can be completed in groups of participants and in a short period of time. 

A paper-based version of the IAT can be a useful measure when looking at implicit attitudes towards people with a disability. Such a measure was developed and validated by Pruett and Chan [4], called the Disability-Attitudes IAT. The Disability-Attitudes IAT measures implicit attitudes towards people with a disability compared to people without a disability. Pruett and Chan [4] demonstrated that able-bodied individuals had greater ease of association between disability symbols and negatively valenced words compared to positively valenced words. These findings suggest that able-bodied adults hold a negative implicit bias towards people with a physical disability. The current study used the Disability-Attitude IAT to examine the prevalence of this negative bias among able-bodied college students.

In addition to establishing the prevalence of stigma towards target groups, IATs can be used to determine whether implicit attitudes toward a single group are moderated by another factor. Moderators are easily examined using this test format because a feature of the IAT is that preference for one concept (e.g., physical activity) is assessed relative to preference for a second concept (e.g., physical inactivity; [21]). Identifying moderators of stigma provides information that can be used to develop stigma reduction strategies. For example, examining moderators may help to identify groups that should be targeted for stigma management interventions or may help to determine the conditions that lessen stigma. 

### 1.3. The Exerciser Stereotype

In a study using measures of explicit attitudes, physical activity status has been established as a factor that reduces the stigma able-bodied people hold towards people with a physical disability [22]. People with a SCI who were described as “exercisers” were viewed more favourably than persons described as “nonexercisers.” This phenomenon is called the exerciser stereotype [23]. This stereotype is pervasive. It has been demonstrated repeatedly in samples of able-bodied adults [23–27] and has been found to create positive impressions of other people, regardless of the age, sex, or activity level of the person rating the exerciser. While findings indicated that stigma can be lessened when people are presented as exercisers, these studies have all been based on explicit attitude measures rather than implicit measures. Often, explicit and implicit attitudes have been found to be unrelated [28]. Therefore, whether the exerciser stereotype exists when measuring implicit attitudes remains unknown.

However, a study by White et al. [29] did examine implicit attitudes towards athletes with a disability. They found that, despite the observation that athletes with a disability are a group that should be least likely to be the target of negative attitudes, as this is a group that people admire for their perseverance and courage [30], attitudes toward this group were consistently negative when compared to able-bodied athletes [29]. While it seems that activity status might not eliminate the stigma of disability entirely, it remains to be determined whether it lessens the stigma experienced.

### 1.4. Study Objectives

The purpose of our study was twofold. First, we aimed to examine the prevalence of negative implicit attitudes towards people with a physical disability. Second, we sought to determine whether the exerciser stereotype measured implicitly reduces the stigma able-bodied adults hold towards people with a disability. In other words, we were interested in determining whether people with a disability were perceived more positively if they were presented as being active versus inactive. 

Consistent with previous research looking at attitudes towards people with a disability [3, 4], our first hypothesis was that the majority of able-bodied participants would view images of people with a disability more negatively than images of those without a disability. The support of this hypothesis would suggest that negative associations between attitudes and disabilities were prevalent. Our second hypothesis was that images of active people with a physical disability would be viewed more positively than images of those perceived as inactive. The support for this second hypothesis would indicate that the exerciser stereotype could be extended to people with a physical disability when measured implicitly, as it would indicate more positive impressions of active people compared to inactive people. 

## 2. Method

### 2.1. Participants

One hundred students from a large university volunteered to participate in this research study (undergraduate: *n* = 81, graduate: *n* = 19). Volunteers were recruited through informational flyers placed in buildings throughout the campus, as well as announcements in courses from all disciplines. In order to participate, volunteers were required to be able to read and understand written instructions in English. The convenience sample included 82 women and 18 men. Participants ranged in age, from 18 years to 36 years old (*M* = 21.48 ± 3.01), and none of the participants had a physical disability. Participants reported whether they had any experience interacting with an individual with a physical disability; approximately how many hours this experience interacting included, and the context of this experience (e.g., volunteer, family member has physical disability). Sixty-five percent (*n* = 65) of participants reported having experience working with people with a physical disability, with a mean number of hours of experience of 153.11 ± 532.17 min. Of the participants that reported having experience working with people with a physical disability, 45% (*n* = 45) indicated that this interaction involved physical activity programming. Informed consent was obtained from all participants, and the study was given ethics approval by the institutional review board. 

### 2.2. Measures

In order to examine implicit attitudes, two versions of a paper-pencil-based IAT were used—the Disability-Attitudes IAT (which included a practice IAT) and the Disability-Activity IAT. Each IAT consisted of two sections, called blocks—a congruent and an incongruent block. The purpose of the practice IAT in the Disability-Attitudes IAT was to familiarize participants with the IAT procedure and was unrelated to disability or physical activity. This practice test instead assessed attitudes towards flowers relative to insects, using words as the stimuli. The category labels on the first block were *flower *and *good* on the left side of the stimulus word, and *insect *and *bad* on the right side. This block was considered to be the congruent block, based on the hypothesis that it would be easier for participants to pair these combinations. The second block, or incongruent block, of the practice IAT showed *flower *and *bad* paired on the left side of the stimulus word, and *insect *and *good* on the right side. In order to complete the test, participants were to categorize each stimulus word as either a flower or insect word, or a good or bad word. Participants were told which words represented insects, which represented flowers, which represented good, and which represented bad in the written instructions they were given prior to the beginning of the test. Participants categorized the words by striking a line through the appropriate circle, either to the left or right of the word. For example, in the congruent block, if the word was a flower or a good word, participants would strike a line in the circle to the left of the stimulus; for insect or bad, they would strike a line through the circle to the right. 

The congruent block of the Disability-Attitudes IAT had *disability *and *bad *paired together and *nondisabled *and *good *paired together. The opposite was true for the incongruent block. The disability/ability-related stimuli were conveyed using stick figure images. The disability images included a diagram of a person with a cane walking, a guide dog, or a pair of axillary crutches, and a stick figure person in a wheelchair. The ability images included a stick figure diagram of a person walking, a person cross country skiing, a stick figure person running, and two stick figure images at a school crossing.

### 2.3. The Development of the Disability-Activity IAT

The Disability-Activity IAT was developed for the present study in order to determine if the exerciser stereotype exists implicitly. The Disability-Activity IAT used picture stimuli rather than word stimuli, consistent with previous measures of implicit attitudes associated with disability [4]. Picture stimuli were chosen over words because it would be very difficult to convey such a complicated concept in only one word. The pictures used in the Disability-Activity IAT were specifically chosen as they were neutral for race, gender and were similar in appearance to each other. It was important that the images were neutral in order to avoid associations with characteristics other than the intended measurement of implicit attitudes. Active images were pictograms developed for the Beijing Paralympic Games and included a stick figure diagram of a person playing wheelchair tennis, a person playing wheelchair rugby, a person playing wheelchair basketball, and a person fencing in a wheelchair. Inactive images included a person in a wheelchair watching television, a person in a wheelchair using a laptop, a person in a wheelchair talking on the phone, and a person in a wheelchair listening to music. Some of these images were changed slightly using Adobe Photoshop to ensure that they were all of the same size and facing the same direction.

**Figure 1:**
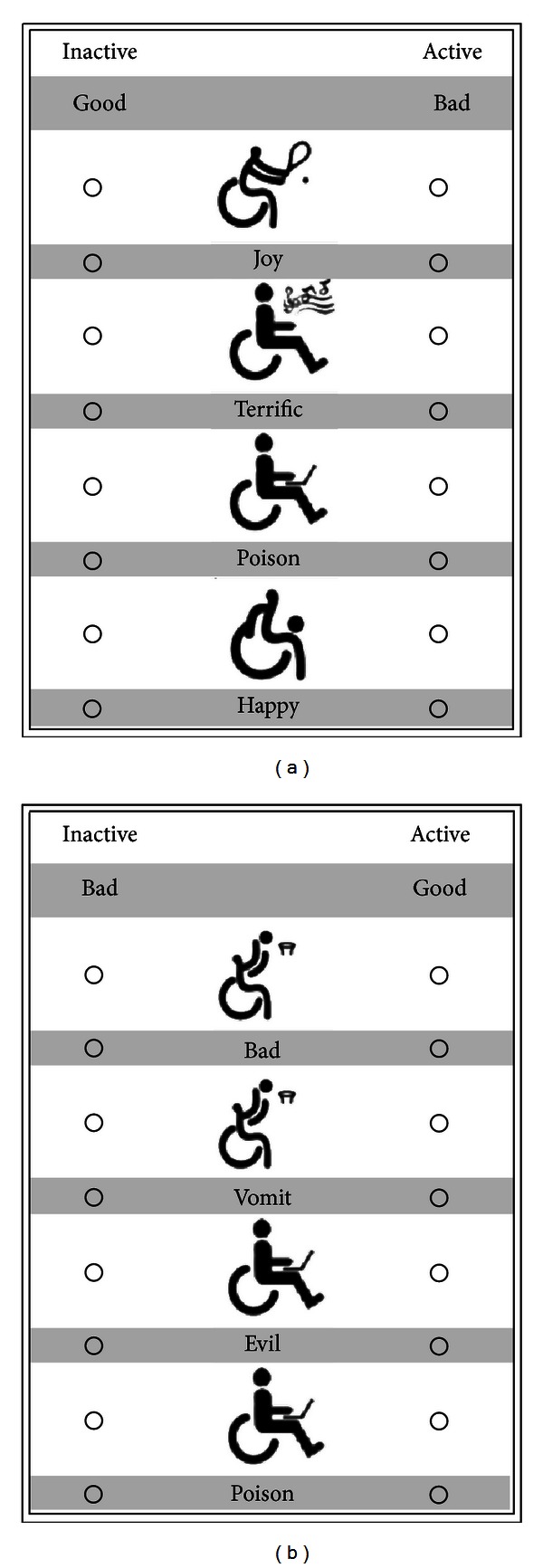
Example of the incongruent (a) and congruent (b) block of the Disability-Activity IAT.

The list of words for the good-bad evaluative category in the Disability-Activity IAT was the same as those used in the flower-insect practice IAT and the Disability-Attitudes IAT. These words were chosen as they were rated high and low in pleasantness in a study by White and colleagues [29], and they were consistent with the other two tests. Good words included terrific, love, happy, joy, and good; bad words included vomit, poison, hatred, evil, and bad. Each page of the Disability-Activity IAT had two rows, with a total of 44 items, consistent with other paper-based IAT tests such as the Disability-Attitudes IAT [4]. Items were alternated between a stimulus (e.g., a picture of a person with a physical disability who was being active or a picture of a person with a physical disability who was inactive) and a good or bad word. Therefore, there were active images, inactive images, good words, and bad words. Both the stimulus words and symbols were randomized on all pages, and no two pages of a given test were identical. In this IAT, the congruent block paired *active *and *good* and *inactive *and *bad. * The incongruent block paired *active *and *bad *and *inactive *and *good. *See Figure 1 for a sample of the Disability-Activity IAT.

### 2.4. Procedure

Participants completed the IAT tests alone or in small groups (2–4 people) sitting at a desk, in a quiet room with the researcher. Before beginning, participants were given written instructions explaining the proceeding tasks. These instructions also indicated that the purpose of the study was to investigate how people organized concepts mentally. Participants were given instructions ensuring that they were unaware of the actual purpose of the study. Each participant then completed six pages of paper-based IATs, each page representing one block of a two-block IAT measure. Therefore, participants completed three IAT measures, which consisted of a practice IAT, the Disability-Attitudes IAT, and the Disability-Activity IAT. The order of the tests remained constant with every participant (practice, and Disability-Attitudes, Disability-Activity); however, in order to counter a possible ordering effect, the order of the blocks of each test was randomly selected. Therefore, some participants completed the incongruent block and then the congruent block of a given IAT, while another participant completed the congruent block followed by the incongruent block. 

Before each IAT, participants read a set of instructions informing them to place their pen in the box at the top of the page before beginning and to complete the first page of the test by starting with the first word in the left column and continuing downward until the entire left column was complete. They could then continue downward starting with the first word in the right column. Participants were informed that they would have 20 seconds to categorize as many words as they could, so they should go as quickly as possible, but try to avoid making mistakes. They were also asked not to correct themselves if they did make an error. After the participant read the instructions, the test administrator then signalled participants to begin the test. The administrator asked participants to stop and to circle the final item they were looking at once the 20 seconds were completed.

Participants were then asked to turn the page and were told that the next task was identical to the last, with the exception that the categories on the top of the page were reversed. This represented the second block of the practice IAT. Following the completion of the practice IAT, participants completed the Disability-Attitudes IAT and the Disability-Activity IAT; the instructions were identical. Before each new IAT test began, participants were given a new written instruction page explaining the next test and which words or symbols belonged in which category. After participants had completed all three tests, they were given a demographic questionnaire to complete. 

### 2.5. Calculation of Scores

The purpose of the practice IAT was simply to familiarize participants with the task. Scores from this IAT were not calculated. In order to calculate the IAT scores of the Disability-Attitudes IAT and the Disability-Activity IAT, first the numbers of correct and incorrect responses were calculated for each block of each test. In cases where more than 35% error was made in any block of either the Disability-Attitudes or Disability-Activity IAT, the responses were deemed invalid. Deeming these cases invalid is consistent with previous studies using paper versions of the IAT, including the study outlining the development of the Disability-Attitudes IAT [4]. The purpose of this step was to remove from the analysis any tests where participants may not have followed directions closely, or where another reason other than having difficulty associating the concepts may have caused a large number of errors.

The score of the two IATs was then computed independently, using the same algorithm used in the development of the Disability-Attitudes IAT [4], which was developed by Nosek and Lane [31]. This algorithm was used as it results in comparable scores to computer-based IATs [4] and is as follows:

(1) ±maximum  value[A,B]minimum  value[A,B]|(A−B)|.

In this algorithm, *A* represents the number of correct responses obtained in the incongruent block and *B* represents the number of correct responses in the congruent block. In occasions where the value of *B* was larger than the value of *A*, the final value obtained was multiplied by −1 in order to restore the meaningful sign of the difference scores lost when using absolute values in the calculation. When the value of *A* was greater than *B*, the values remained unchanged. This transformation was applied to the Disability-Attitudes IAT only. Thus, for the Disability-Attitudes IAT, a final result with a negative value suggested negative attitudes towards people with a disability compared to people without disabilities. Conversely, if the final value was positive, this was representative of positive attitudes toward people with a disability in relation to people without a disability. A score of zero suggested that attitudes towards people with a disability were equal to the attitudes towards people without a disability, representing no stigma. 

With the application of this algorithm to the Disability-Activity IAT, a positive score indicated positive attitudes towards people with a physical disability who were active compared to inactive, and a negative score reflected negative attitudes towards active people with a physical disability compared to those who were inactive. A score of zero on the Disability-Activity IAT indicated that attitudes towards inactive people were equal to those toward active people, or that no bias existed.

### 2.6. Data Analyses

#### 2.6.1. Scale Validity

In order to assess the validity of the scales, descriptive statistics were calculated for each scale to identify the frequency of correct and incorrect responses for both blocks of each test. A paired sample *t*-test was then used to determine if there was a significant difference between the number of correct congruent responses and the number of correct incongruent responses. Similarly, a paired sample *t*-test was used to determine if there was a significant difference between the number of errors in the incongruent block and the congruent block. These analyses were used to test the validity of scales, as done by Pruett and Chan [4] to validate the Disability-Attitudes IAT.

#### 2.6.2. Hypothesis Testing

In order to assess the first hypothesis that images of people with a disability would be viewed more negatively than those without a disability, analyses were conducted using the Disability-Attitudes IAT. An independent sample *t-*test was performed to determine if the scores (obtained using the previous algorithm) were significantly different than zero. The results were compared against the value of zero, as this is the value associated with no bias.

 In order to evaluate the second hypothesis that active people with a physical disability would be viewed more positively than inactive people with a disability, analyses were conducted using the Disability-Activity IAT. An independent *t*-test was performed to determine if the scores were significantly different than zero. In this case, zero was the value used to indicate the absence of the exerciser stereotype (i.e., that people with a physical disability who are active and inactive are viewed equally). 

## 3. Results

Following the removal of cases where participants made more than 35% error in one of the four blocks of Disability-Attitudes IAT and Disability-Activity IAT (20 cases), eighty participants provided both a Disability-Attitudes IAT and the Disability-Activity IAT that were deemed valid for analysis. 

### 3.1. Disability-Attitudes IAT

#### 3.1.1. Scale Validity

Preliminary analyses of the Disability-Attitudes IAT indicated that participants were able to make significantly more correct congruent associations (*M* = 17.59, SD = 4.81) compared to incongruent associations (*M* = 12.48, SD = 3.75), *t*(79) = −10.24, *P* < 0.01. Participants, however, did not make significantly more errors in the incongruent block of the test (*M* = 1.18, SD = 1.55) than in the congruent block (*M* = 0.84, SD = 1.27), although there was a trend indicating that more errors were made in the incongruent block, *t*(79) = 1.843, *P* = 0.07.

#### 3.1.2. Hypothesis Testing

**Figure 2:**
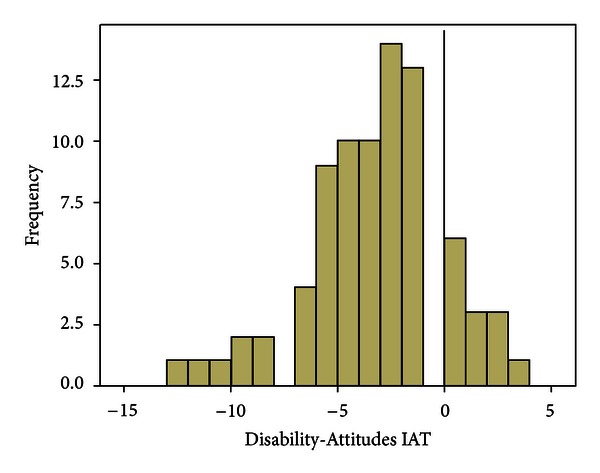
Frequency of Disability-Attitudes IAT scores. Vertical line represents a score of zero, the score associated with no bias towards people with a physical disability. Negative scores represent negative attitudes towards people with a physical disability and positive scores represent positive attitudes towards people with a physical disability.

The first hypothesis that people with a disability would be viewed more negatively than persons without a disability was tested using the results from the Disability-Attitudes IAT. The application of the scoring algorithm produced a negative Disability-Attitudes IAT score (*M* = −3.33, SD = 3.07), which was significantly different than zero (the value associated with no bias), *t*(79) = −9.72, *P* < 0.01. This was consistent with the first hypothesis. The frequency of these scores can be seen in Figure 2. The standardized effect size, Cohen's *d*, was calculated at 1.09, indicating a large effect [32]. A total of 83.8% of participants had a negative composite score and exhibited negative attitudes towards images of people with a disability. Approximately, 7.5% of participants exhibited no bias and 9.7% exhibited positive attitudes towards the images of people with a disability.

### 3.2. Disability-Activity IAT

#### 3.2.1. Scale Validity

Preliminary analyses of the results of the Disability-Activity IAT revealed that participants were able to make significantly more correct congruent associations (*M* = 22.16, SD = 5.09) than incongruent associations (*M* = 12.89, SD = 3.27), *t*(79) = −19.14, *P* < 0.01. Furthermore, the frequency of error of incongruent associations (*M* = 0.75, SD = 1.04) was significantly higher than congruent associations (*M* = 0.26, SD = 0.57),  *t*(79) = 3.73,   *P* < 0.01.

#### 3.2.2. Hypothesis Testing

**Figure 3:**
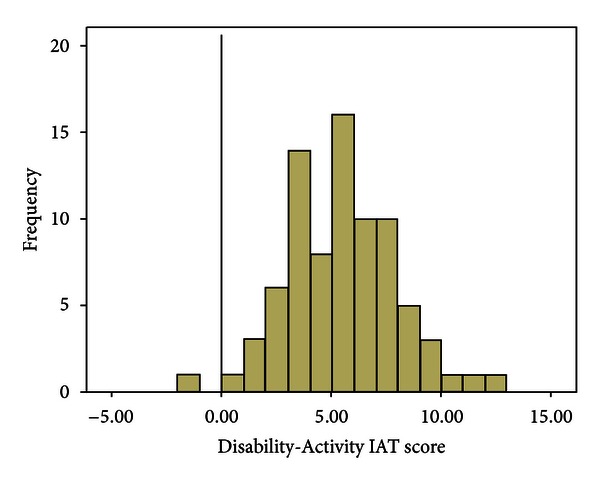
Frequency of Disability-Activity IAT scores. Vertical line represents a score of zero, the score associated with no exerciser stereotype. Negative score represents negative attitudes towards people with a physical disability who are inactive positive score represents positive attitudes towards people with a physical disability who are active.

The second hypothesis that active people with a physical disability would be viewed more positively than inactive people with a physical disability was tested using the results from the Disability-Activity IAT. Consistent with the second hypothesis, the application of the scoring algorithm produced a positive Disability-Activity IAT score (*M* = 5.48, SD = 2.57), which was significantly different than zero (the value associated with no exerciser stereotype), *t*(79) = −19.09, *P* < 0.01. The frequency of these scores can be seen in Figure 3. The standardized effect size, Cohen's *d*, was calculated at 2.13, indicating a large effect [32]. A total of 97.5% of participants had a positive composite score and exhibited positive attitudes towards images of people with a physical disability who were active. A total of 1.3% of participants exhibited no bias and only 1.3% exhibited negative attitudes towards the images of people with a physical disability who were active.

## 4. Discussion

 This study examined the implicit attitudes of able-bodied individuals towards people with a physical disability compared to those without a disability. Furthermore, the study tested whether or not the exerciser stereotype existed for people with a physical disability such as SCI when assessed using an implicit measure. The results of the study confirmed the two hypotheses, indicating that the majority of participants had negative implicit attitudes towards people with a disability and particularly people who were inactive. These findings demonstrate that stigma does appear to exist towards people with a physical disability, as supported by previous health-related stigma literature [3]. The results of this study also provide further evidence to support the idea that people with a physical disability may be able to use information regarding their exercise habits as an effective strategy to minimize, or manage, the stigma they are faced with, due to their disability. Future research, however, is still needed to understand the extent that this stigma management occurs, or the extent to which stigma is lessened, when people with a physical disability provide information about their participation with exercise. 

Consistent with previous research in the general population, and for people with a SCI [22], which measured the exerciser stereotype explicitly, the results of this study demonstrate that the stereotype can be extended to implicit measures of attitude as well. This finding adds further evidence that suggests that by providing information about one's exercise or physical activity participation, people with a physical disability can reduce the negative impressions that are formed towards them by able-bodied people. This finding can be used as an effective way to manage the stigma people with a physical disability are so frequently faced with from able-bodied people. 

This study was an important step in understanding stigma towards people with a physical disability and in determining that implicit stigma can be moderated by physical activity. This IAT demonstrated convergent and discriminant validity such that participants in the study made significantly more correct congruent associations than incongruent associations. As well, participants made more errors during incongruent association than congruent providing evidence on how the Disability-Activity IAT measures the implicit attitudes. According to Pruett and Chan [4], more errors should be made during the incongruent association task than the congruent association task if the instrument actually measures ease of evaluative association. Therefore, it appears that the Disability-Activity IAT measured congruent and incongruent associations consistent with automatic evaluation of physical activity-related and physical inactivity-related symbols of people with a physical disability. 

While the results from our study offered support for our hypotheses, several limitations should be addressed in future research. Firstly, the sample studied may not be generalizable to the general population, as a large percentage of the student participants had had experience working with an individual with a physical disability. In the general population, there may be more individuals who have little to no exposure to individuals with a disability. This difference in exposure may influence attitudes. Furthermore, in the current study, we were not able to determine if the exerciser stereotype existed because more positive impressions were formed of active people, or because more negative perceptions were formed of inactive. Alternatively, the stereotype might have resulted from a combination of these impressions. To address this limitation, future studies should also further assess attitudes towards people with a physical disability where no indication of activity is given. In addition, though paper-based IATs have been shown to be a valid measure of implicit attitudes towards group, they do not have sufficient reliability to determine the attitude of one individual person [4]. Therefore, future studies use a computer-based version of the Disability-Activity IAT, which would provide sufficient sensitivity to measure individual attitudes. 

The use of picture-based stimuli was a limitation for two reasons. First, picture-based IATs tend to elicit smaller effects than word-based IAT [4]. However, this limitation is difficult to address because it is difficult to describe a person with a disability in a single word and even harder to explain a person's activity status. Second, the Disability-Attitudes IAT included activity-based images, most of which were used for the able-bodied stimuli. These activity-based stimuli may have inadvertently created an exerciser stereotype favouring the able-bodied stimuli. This confound may have led to an overestimation of the prevalence of stigma towards people with a disability. 

Despite these limitations, this study helps to advance our understanding of stigma towards people with a physical disability and provides direction for future research. The study findings suggest that a paper-based IAT can be used to measure implicit attitudes towards people with a disability. It also can be used to examine factors that might moderate these attitudes. Moreover, the study highlights the prevalence of negative implicit attitudes towards people with physical disabilities and suggests that participating in exercise might help to mitigate this stigma.
